# Analysis of Gene Expression Changes in Plants Grown in Salty Soil in Response to Inoculation with Halophilic Bacteria

**DOI:** 10.3390/ijms22073611

**Published:** 2021-03-31

**Authors:** Ashley K. Miller, Brent L. Nielsen

**Affiliations:** Department of Microbiology & Molecular Biology, Brigham Young University, Provo, UT 84602, USA; miller.ashley.kay@gmail.com

**Keywords:** halophiles, plant growth-promoting rhizobacteria (PGPR), RNA sequence analysis (RNA-seq), quantitative reverse transcriptase PCR (qRT-PCR)

## Abstract

Soil salinity is an increasing problem facing agriculture in many parts of the world. Climate change and irrigation practices have led to decreased yields of some farmland due to increased salt levels in the soil. Plants that have tolerance to salt are thus needed to feed the world’s population. One approach addressing this problem is genetic engineering to introduce genes encoding salinity, but this approach has limitations. Another fairly new approach is the isolation and development of salt-tolerant (halophilic) plant-associated bacteria. These bacteria are used as inoculants to stimulate plant growth. Several reports are now available, demonstrating how the use of halophilic inoculants enhance plant growth in salty soil. However, the mechanisms for this growth stimulation are as yet not clear. Enhanced growth in response to bacterial inoculation is expected to be associated with changes in plant gene expression. In this review, we discuss the current literature and approaches for analyzing altered plant gene expression in response to inoculation with halophilic bacteria. Additionally, challenges and limitations to current approaches are analyzed. A further understanding of the molecular mechanisms involved in enhanced plant growth when inoculated with salt-tolerant bacteria will significantly improve agriculture in areas affected by saline soils.

## 1. Introduction

### 1.1. Soil and Crop Loss Due to Rapidly Increasing Concentrations of Salinity Buildup in Soil

According to the U.S. Department of Agriculture (USDA), about 10 million hectares of land are lost yearly to soil salinization. The rate of soil salinization is influenced by many factors, including the practice of poor irrigation techniques and local climates. In arid climates such as Utah, the rate of salinization has been documented to occur about three times faster than in temperate climates [1]. Most plants do not have the biochemical or structural mechanisms needed to survive in high salt environments, leading to decreased plant yield.

Salinity causes several negative effects on plant growth and development due to water stress, cytotoxicity from elevated levels of sodium and chloride ions in the cytoplasm, nutritional imbalance, the production of ethylene, reactive oxygen species and other oxidative damage. These disruptions can seriously affect plant productivity [1,2]. Unless these stressors are at least partially alleviated, severe decreases in plant yield can result.

### 1.2. Limitations of Bioengineering Salt Tolerance

As improving plant salt tolerance is of mounting importance around the world, a number of approaches have been taken to address this problem. Introducing genes to confer salinity tolerance to plants is one approach. It has been reported that transformed tobacco plants expressing the gene for ectoine showed improved growth in salty soils [3]. Others suggest that *Medicago truncatula* production could be improved in salty soils when transformed with the rstB gene [4]. Some native plant genes have also been shown to play a role in plant salt tolerance, such as PPR40 in Arabidopsis [5], ethylene response factors [6], K+ transporter genes [7], and the chimeric ryegrass gene OsDST-SRDX, among others [8]. Still, there is much that is not understood about plant salt tolerance. For example, it is likely that multiple genes may be needed to provide meaningful increases in salinity tolerance in transgenic plants, and different genes may be required for different species. In addition, transcriptional control elements may also differ between species [9]. These and many more questions still need to be addressed in greater depth.

There are other potential limitations outside of genetic engineering. One of these limitations is the approval process. In the United States there are three federal agencies that oversee the approval of bioengineered crops: (1) the Food and Drug Administration (FDA), (2) the Environmental Protection Agency (EPA), and (3) the U.S. Department of Agriculture (USDA). Each of these agencies must be involved in the approval process of new genetically modified organisms (GMOs) [10]. The approval process is often long and difficult. There is one additional and significant limitation to a GMO salt-tolerant crop, and that is the public perception. In an age where the safety of animal feed and the direct human consumption of GMOs is questioned and avoided by many, public perceptions of bioengineered salt-tolerant crops could potentially limit their use and impact [11].

### 1.3. Survival Skills: How Halophytes Survive in Salty Soil

While most plants lack necessary mechanisms of salt tolerance, there are uniquely adapted plants named halophytes that complete their entire life cycle in soils containing 200– 500mM salt [1]. There are four main mechanisms of halophyte salt tolerance including: (1) the secretion of salt through salt glands, (2) the regulation of cellular ion homeostasis and osmotic pressure, (3) the detoxification of reactive oxygen species, and (4) alterations in membrane composition [6,7,12,13,14,15]. Each mechanism listed above is energetically expensive. As a result, most plants will need to increase photosynthesis to maintain homeostasis under saline conditions [1,12,13,14,15].

**Figure 1:**
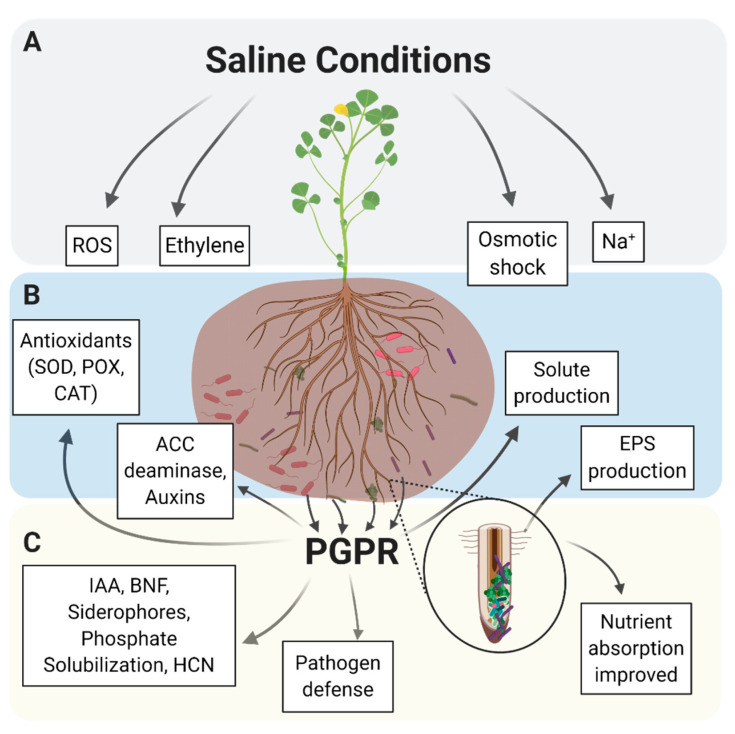
Salt tolerance-related plant growth promoting rhizobacteria (ST-PGPR) and their role in enhanced salt tolerance in halophytes. (**A**) Saline soils cause an abundance of toxic molecules such as ethylene and reactive oxygen species (ROS) to form and impede plant processes, leading to disease and death in plants without sufficient mechanisms of salt tolerance. Na^+^ ions disrupt the function of plant ion channels, leading to plant osmotic shock. (**B**) Direct mechanisms by which ST-PGPR can enhance plant salt tolerance. Each ST-PGPR produces different antioxidants or solutes to help fight salt toxicity. (**C**) Indirect methods of ST-PGPR plant salt tolerance enhancement. Not all ST-PGPR rhizobium produce all substances mentioned above. Image produced based on ideas adapted from [15].

However, plants are not alone in their battle against increasing salinity. The plants’ microbiota also play an important role in overall salt tolerance (see Figure 1; effects shown in B–C).

## 2. The Role of Halophilic Plant-Associated Bacteria in Plant Growth under Saline Conditions

Halophyte microbiomes contain plant growth promoting microbes both within the plant (endosymbionts) and outside the plant around their root system (ectosymbionts). These symbionts are often involved in promoting plant growth under stress and are referred to as plant growth promoting rhizobacteria (PGPR). Some strains also assist halophyte survival under salty conditions; such salt tolerance-related PGPR are noted as ST-PGPR [16]. ST-PGPR often fall into one of the salt-loving genera such as *Halomonas* and *Halobacillus*, though microbes from the genus *Kushneria* and other genera have similarly been shown to promote salt tolerance [1].

In recent years, there has been a significant increase in the number of publications that demonstrate the ability of ST-PGPR to improve salt-sensitive (glycophyte) crop yield in saline soil [1,14,16,17,18,19]. Many of these glycophytes are members of the grass family including rice, wheat, and barley, though similar success with legumes including soybean have been reported [19,20,21]. In rice, it was reported that two strains of endemic *Halobacillus* including *Halobacillus dabanensis* strain SB-26 and *Halobacillus* sp. GSP-34 greatly increased salt tolerance and plant yield when inoculated into saline soils [2]. Similarly, in wheat, ST-PGPR including *Halomonas* sp. demonstrated a 62.2% to 78.1% increase in the length and wet mass of root and shoot tissues when compared to plants grown in the same salty soil but without inoculum [18]. Nakayama et al. [3] showed that *Halomonas elongata* could be used to improve the salt tolerance of tobacco plants. Similarly, ST-PGPR strains from the microbiome of native Utah halophytes, when added to salty soil containing non-salt-tolerant *Medicago sativa* (alfalfa) seedlings, reportedly increased plant yield when compared to controls [1]. Some of these ST-PGPR strains were isolated from the rhizosphere closely associated with roots, while other bacteria were isolated from within plant tissue.

### 2.1. Potential Mechanisms for Plant Growth Stimulation by ST-PGPR

Mechanisms by which endophytes enhance plant growth are thought to include improved nutrient acquisition and changes in gene expression. For example, ACC (1-aminocyclopropane-1-carboxylase) deaminase is a bacterial enzyme found in many endophytes that stimulates nutrient acquisition and plant growth by reducing the amount of ACC converted to ethylene. Ethylene is a known inhibitor of plant growth that is produced by the plant in response to salt, drought, and other environmental stresses [16,22]. *Burkholderia phytofirmans* is another endophyte that alters plant gene expression to enhance the growth of multiple cultivars of switchgrass [23,24]. Inoculation with this strain was found to induce wide-spread gene expression changes in the plant host, including the altered expression of some transcription factors that are known to regulate the expression of plant stress genes [25]. Other bacterial endophytes (species of S*phingomonas, Pantoea, Bacillus* and *Enterobacter*) have been identified as enhancing the salt tolerance of hybrid elephant grass [26], likely because of enhanced nutrient acquisition and/or gene expression changes. Plants inoculated with *Halomonas elongata* (accession number MK873884) and 1% NaCl demonstrated an average increase of 2.4-fold in the biomass of alfalfa plants grown without inoculum [1]. These data suggest that this strain of *Halomonas* influences plant salt tolerance, raising the question of how these bacteria stimulate plant growth under saline conditions. *H. elongata*, a moderate halophile, produces a well-studied osmolyte named ectoine. Ectoine is produced by the bacteria in direct relationship with how much salt is in the environment surrounding the microbe. As levels of salt in the environment increase, so do the ectoine levels in the bacteria. This increase in ectoine helps the bacteria regulate osmotic pressure and fend off salt toxicity [3,16,17,19]. Though it remains unclear whether ectoine is among or is the mechanism of observed salt tolerance conferred from *H. elongata* to plants, there is growing evidence that ectoine plays some role in improving salt tolerance. A study with tobacco plants showed that significant salt tolerance did occur in plants inoculated with *H. elongata* producing ectoine vs. an ectoine knockout mutant; plants inoculated with the knockout mutant showed a reduction in salt tolerance [3].

The development, understanding, and application of well-characterized halophilic bacteria isolated from halophytes could help alleviate many of the challenges of soil salinity, without the use of genetic engineering. Additionally, since these isolates are in their native state, using them to inoculate plants would be considered an organic treatment. PGPR technology has been applied to crop production for decades [27,28]. The focus in this review is on the effects of ST-PGPR inoculation on gene expression in the plants that lead to enhanced plant growth in salty soils.

### 2.2. Changes in Plant Gene Expression in Salt-Grown Plants Inoculated with ST-PGPR

The stimulation of plant growth in salty soil, in response to inoculation with halophilic ST-PGPR, infers that the expression of at least some plant genes are altered to enhance plant growth. In general, glycophytes exhibit a significant decrease in photosynthesis when exposed to salinity stress, along with an increase in the production of reactive oxygen species (ROS). One mechanism by which ST-PGPR stimulate plant growth is to offset the decrease in photosynthesis by stimulating the expression of proteins involved in this process in the plant [29,30]. One such ST-PGPR stimulated gene is auxin, a plant hormone involved in regulating growth. Increased auxin production was reported for *Salicornia* plants inoculated with actinobacteria and irrigated with seawater [29]. Taj and Challabathula [30] observed an increased rate of photosynthesis and a decrease in ROS in both tomato and rice inoculated with *Staphylococcus sciuri*.

Some other plant gene expression pathways likely to be involved are those that regulate redox potential, ion homeostasis, leaf gas exchange, ion transport, osmolyte production and other genes involved in the stress response. Several studies have shown that inoculation with halotolerant bacteria enhances plant growth by inducing increased expression of antioxidant enzymes and proteins. For example, an increase in plant osmolyte production has been shown to occur in tomatoes inoculated with *Streptomyces* [31], *Staphylococcus sciuri* [30], *Limonium* (a coastal halophyte) and *Bacillus flexus* [32]. Several of these genes are components of stress response pathways. Transcription factors that control these pathways are also likely differentially expressed in inoculated plants exposed to salinity stress. In maize and chickpea, some genes involved in these pathways have demonstrated differential expression in response to microbial inoculation [33,34].

Bharti et al. [35] showed that several genes involved in salinity tolerance were differentially expressed in both roots and shoots of wheat in response to inoculation with a strain of *Dietzia natronolimnaea* and growth of the plants in salty soil. Their studies identified the role of an abscisic acid (ABA)-signaling cascade which led to the upregulation of TaABARE and TaOPR1 and the induction of TaMYB and TaWRKY transcription factors, leading to the stimulation of several stress-related genes. In addition, they found that TaNHX1, TaHAK and HaHKT1 ion transporter genes were differentially expressed in these plants, while antioxidant enzymes were upregulated under these same conditions.

A similar study with wheat inoculated with *Arthrobacter nitroguajacolicus* was conducted by Safdarian et al. [36]. This study involved the comparative transcriptomic analysis of un-inoculated and inoculated wheat roots under salt stress. They found that 152 genes were significantly upregulated, and five genes were downregulated under these conditions. Many of the upregulated genes are involved in antioxidant biosynthesis, flavonoid, porphyrin, and chlorophyll metabolism.

Another study showed the increased expression of two ZmPIP (plasma membrane aquaporin protein) isoforms in response to inoculation with ST-PGPR [37]. ST-PGPR reportedly induce osmolyte accumulation and phytohormone signaling, allowing plants to overcome osmotic shock caused by salinity. The inoculation of halophilic *Bacillus amyloliquefaciens* SN13 onto rice (Oryza sativa) plants enhanced salt tolerance when the rice was exposed to salinity (200 mM NaCl). The expression of 14 genes were affected by the following: SOS1, ethylene responsive element binding proteins EREBP, somatic embryogenesis receptor-like kinase SERK1, and NADP-malic enzyme (NADP-Me2). In the presence of these elements, 14 genes experienced upregulation, while two (glucose insensitive growth GIG and (SNF1) serine-threonine protein kinase SAPK4) were downregulated in plants grown hydroponically in response to salinity [37].

Some studies have used the model plant *Arabidopsis thaliana* to examine differential gene expression in response to inoculation with salt-tolerant bacteria. Baek et al. [38] showed that plants inoculated with *Bacillus oryzicola* exhibited increased chlorophyll production and the activation of the SOS1-dependent salt signaling pathway in comparison to uninoculated plants. SOS1 is a plasma membrane-localized Na^+^/H^+^ antiporter. Liu et al. [39] performed transcriptome profiling in Arabidopsis inoculated with *Bacillus amyloliquefaciens*, resulting in 1024 upregulated and 264 downregulated genes in plants grown in 100 mM NaCl compared to uninoculated plants grown at the same salinity. Upregulated plant genes included those involved in auxin-mediated signaling, SOS scavenging, sodium ion transport, photosynthesis, and the production of osmoprotectants, including trehalose and proline. They also analyzed hormone pathway mutants and determined that ethylene/jasmonic acid signaling but not ABA signaling may be altered in inoculated plants to increase their salt tolerance.

## 3. Overview of RNA Sequencing and Data Analysis

The regulation of gene expression is not just an on/off switch but can be better compared to the volume dial on a radio, where expression can be dialed up or dialed down. In the cell, the gene expression dial is adjusted according to environmental change and cellular need [40]. Thanks to next generation sequencing (NGS) and bioinformatics, it is easier than ever to analyze the volume of gene expression [41]. Gene expression analysis is a multi-step process with two main parts: 1) sequencing, and 2) analysis of sequence data [42]. In the studies discussed below, we focus on the analysis of differential gene expression in response to the inoculation of plants with halophilic bacteria, compared to uninoculated plants, grown in salty soil. Both sequencing and analysis necessitate multiple sub-steps which will be briefly described.

**Figure 2:**
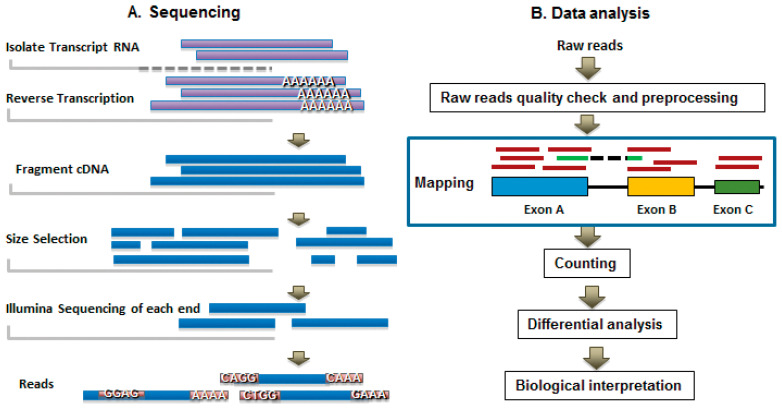
General overview of the steps involved in RNA- sequencing and subsequent analysis. (**A**) The major steps of RNA processing, sequencing, and read output. (**B**) Main steps involved in RNA- sequence analysis. Figure from “Bioinformatics for RNA- Seq Data Analysis” opensource article [42].

Sequencing begins with the isolation of plant RNA, which is critical for all subsequent steps. RNA is a notoriously unstable molecule due to its single-stranded nature and 2′ hydroxyl group on the ribose sugar, making the RNA molecule vulnerable to degradation. This means that isolated RNA samples must always be kept on ice when working in the lab and at −80C when stored. Careful isolation also includes avoiding contamination by anything that contains RNase or will inhibit downstream steps [43]. Plant RNA can be purified using a Trizol method or using an RNA isolation kit such as from Qiagen or Invitrogen, coupled with using a shredder spin column to improve RNA recovery from plant tissue. Following isolation, the RNA undergoes a quality assessment by Qubit or High Sensitivity Fragment Analysis to ensure that the RNA is intact and to determine its concentration. Once RNA quality has been assessed and is acceptable, the next step is reverse transcription to produce cDNA, which is significantly more stable than RNA. At this point, two options present themselves: (1) perform library prep for sequencing in lab, or (2) send cDNA to a sequencing center for further processing [44]. Either option will require the cDNA to undergo three additional processing steps: (1) cDNA fragmentation, (2) size selection of cDNA fragments, and (3) NGS on the appropriate instrument (See Figure 2A). The output of NGS is a large file of raw reads which represent base pairs inferred by the sequencer during processing. After processing, reads are then quantified and analyzed using bioinformatic tools.

As with all data analysis, the first step is to check quality. Since adapters are ligated onto the cDNA during library prep, it is important to remove these sequences before further processing [45]. Here, reads are mapped to an annotated transcriptome. If the plant of interest has an annotated transcriptome this step is rather simple and straightforward. If the percent of reads mapped is low, it is wise to re-evaluate the earlier quality control steps. If the percent of reads mapped are high, then generally moving on to further analysis is permissible. Moving on, however, requires the answer to the question, “which genes are differentially expressed among samples?” Uncovering the answer is assisted through building comparative table(s) and plotting data to help visualize fold changes in gene expression. Further, performing gene ontology analysis is another great tool for understanding gene function and potential interaction pathways (See Figure 2B).

Unfortunately, not every model organism has an assembled transcriptome, let alone a well annotated transcriptome. What then? Thanks to bioinformatic packages and assemblers such as trinity and SOAPdenovo-trans, there are many ways to create a de novo assembly [46]. A de novo assembly, however, would not have any gene annotation. For this reason, de novo assemblies are not very informative on their own. If, however, there is a related organism with an annotated transcriptome, the de novo transcriptome can be compared with that of its relative. This comparative analysis helps to determine gene name, function, expression levels, and potential ontologies. This process is generally referred to as “lift over” and will be further described in the next section. This next section outlines some of the approaches used to analyze differential plant gene expression in response to the bacterial inoculation of alfalfa plants grown in the presence or absence of salt.

### 3.1. Approaches for Analyzing Changes in Plant Gene Expression in Response to Bacterial Inoculation

All approaches used to determine differentially expressed genes (DEGs) in plants start with a well-designed growth trial. Alfalfa grown with or without the inoculation of *Halomonas elongata* (accession number MK873884) under four soil conditions: (1) no salt or bacterial inoculant, (2) salt (1% NaCl) without bacteria, (3) bacteria without salt, and (4) bacteria and salt assist in the analysis of gene expression changes for each growing condition. After about 8 weeks of growth, plants can be harvested for root and shoot tissues. These tissues can help determine whether differential gene expression occurs, and if so, which of the four growing conditions and/or tissue types the gene expression change is associated with. In order to assess the tissues for DEGs, RNA needs to be extracted. One option is to grind tissues samples individually in liquid nitrogen for total RNA isolation using a Purelink RNA Mini kit with a homogenizer for plant tissue (Invitrogen). After grinding, tissue samples from each growing condition are lysed and homogenized. The lysate is then passed through a spin column to remove genomic DNA and washed to remove contaminants. Total RNA is then eluted from the column and subjected to quality testing, often performed on a Bioanalyzer. Quality RNA is then converted into a library of cDNA, which is either sequenced in house or sent to a sequencing center.

Sequence data files (reads) are subsequently reviewed for quality using a package such as FASTQC. Sequence reads are then aligned to the *M. sativa* transcriptome on a bioinformatics platform such as R. If using R, Rsubread is a robust splice-aware package that will align, quantify, and analyze RNA-seq data. After alignment, genes and their associated quantifications are determined for each condition and tissue type bioinformatically. Due to the incomplete nature of the alfalfa gene ontology (GO) annotation, converting alfalfa genes to their most similar homolog in *Arabidopsis*, in a process generally referred to as “lift over,” can improve expression analysis. The results of the GO analysis, when collected into a super-table containing all results from each tissue type and growth condition, allows for easy visualization and comparison among different growth conditions and tissues.

### 3.2. Mining Sequence Data for Candidate Genes

As previously mentioned, the inoculation of *Halomonas elongata* (accession number MK873884) demonstrated an increase in alfalfa growth under salt-stress when compared to the control. From this observation developed the hypothesis that the increase in plant growth was likely due to changes in plant gene expression induced in the presence of the bacterial inoculum [1]. To test such a hypothesis careful analysis of RNA-seq data representative of each growing condition and plant tissue type must be acquired. Once tables representing all data types are produced, candidate features are easily isolated via the quick-and-dirty method of table sifting in Excel, or more eloquently, through bioinformatics. Candidate features should represent both significantly up- and downregulated genes with low Q-values (i.e., <1 × 10^−5^). A Q-value is a false discovery rate (FDR) adjusted p-value. This value adjusts the original p-value to increase statistical stringency and further reduce the presence of false-positive results. The lower a given Q-value, the more significant a feature (i.e., up- or downregulated gene) is within the study [47]. Once gene candidates are identified, it is common practice to validate targets by performing quantitative reverse transcriptase PCR (qRT-PCR) [48]. To do so, RNA is isolated (as before described) from root and shoot tissue of alfalfa grown in the presence or absence of the bacterial inoculum and salt. Then, cDNA is prepared from isolated RNA using the Thermo Fisher Superscript IV. With the sample now prepared, primers specific to the gene candidates are designed using the analysis results from RNA-seq data. To determine potential changes in gene products among different alfalfa growth treatments and tissue types, qRT-PCR is used to quantify gene candidate amplification. The ABI StepOnePlus Real-Time PCR System is one of the commonly used instruments to carry out this process. There are two methods of measuring qPCR amplifications: 1) by an intercalating dye, or 2) via a fluorescently labeled probe [49]. Intercalating dyes are generally the more cost-efficient method of qPCR amplification when many gene targets are being verified. Probes, however, are generally considered the cleaner and more simplistic mode of quantification, as they generally require less optimization. One example of a commonly used intercalating dye for qPCR is the standard PowerUp SYBR Green Master Mix. One important note about SYBR is that it binds to any double stranded DNA, including primer–dimers [50]. As a result, melt curves should always be reviewed for each well after qPCR. If more than one melting curve presents, it may be due to primer–dimer amplification. One way to confirm the correct product and absence of primer–dimers is by analyzing qPCR products via gel electrophoresis. Gel visualization is an excellent tool for testing if multiple melting curves are associated with more than one amplicon. If the gel returns only one band then likely the multiple melting curves shown on the qPCR machine are artifacts of intermediate steps in the amplification process and are not due to primer–dimer formation. Alternatively, programs such as uMelt curve prediction software (a free online tool) can accurately predict melting curves. This type of predictive software serves as a comparative tool against the melting curve produced by the qPCR instrument [51]. As always, selecting an appropriate control transcript as reference, generally a house keeping gene such as the 18S rRNA transcript, is critically important. For the amplification curve and general results, the 2^-ΔΔ*C*T^ method is selected for comparison. Biological and technical replicates (three for each) are generally worked into the qRT-PCR set-up, improving confidence in and analysis of later results.

### 3.3. Limitations of RNA Sequencing and Analysis

Before exploring the limitations of RNA sequencing and analysis, it is appropriate to clearly state that these technologies have been revolutionary and their impact critical in many technological and procedural advances [42]. Even so, these tools have limitations and weaknesses that have not yet been overcome.

One such limitation to the success of RNA-seq and analysis is user error during library preparation [52]. For instance, one important factor in good library preparation for RNA-seq is the size selection of cDNA fragments [53]. Selecting the correct size fragments improves the odds that most low molecular weight dimers are not included in sequencing, which improves the efficiency and accuracy of the sequence analysis [54]. Similarly, sequencing depth must be sufficient to cover low-abundance transcripts multiple times. Doing so improves sequencers’ base pair inference, which also helps to improve sequencing accuracy [55].

Further, commonly used second-generation sequencers (i.e., Illumina) have some serious flaws. Short reads produced by this generation of instruments can become a problem if performing a de novo assembly is desired [56]. Short read sequencing (SRS) uses fragmented DNA generally 75–400 bp long. These short read lengths can become a problem as the size of the genome being sequenced increases. For instance, larger complex genomes such as that of humans contain many repetitive sequences, and due to the nature of PCR, these sequences are preferentially amplified. As preferential amplification increases, there is a risk of failing to generate enough sequence overlap between fragments for a quality de novo assembly. This lack of sequence overlap makes stitching scaffolds into one contiguous sequence very difficult, as an incredibly high number of reads are produced during short read sequencing [57].

Another common limitation to RNA-seq analysis is understanding bioinformatic packages well enough to select the best fit. For instance, three common comparative genomics aligners are: (1) Bowtie2, (2) TopHat, and (3) Rsubread [58,59]. All three of these tools serve to align sets of reads to a reference genome, but each does so with different strengths and weaknesses [60]. Bowtie2, while especially good at aligning large sets of reads to a reference genome, often fails to capture rare transcripts. TopHat on the other hand, is a tool that builds upon Bowtie2, improving the splice variant problem and reliably capturing rare transcripts [61]. Additionally, Rsubread is a package with robust read mapping of both small and large genomes and performs quantification and variant analysis [59]. Though a great option, Rsubread requires some understanding of the R platform and language to use packages such as Rsubread [62].

## 4. Conclusions and Future Challenges

The potential for using salt-tolerant plant-associated bacteria as inoculants of glycophyte crop plants to enhance growth and yield is clear from an increasing number of reports in the literature. However, understanding the mechanisms driving plant growth stimulation by these bacterial inoculants is currently limited. Photosynthesis is reduced in plants under salinity stress, while inoculation with ST-PGPR often results in increases in photosynthesis, as discussed in this review. Clearly, the expression of many genes is altered in the inoculated plants, resulting in plants with an increased tolerance to salinity and enhanced growth. While additional potential bacteria and plant interactions need to be studied, it appears that there may be some bacterial species–plant specific interactions, making it difficult to make general conclusions about the mechanisms involved. The properties of the bacterial inoculant may directly provide some help in reducing the effect of salinity by stimulating photosynthesis (see Figure 1), but different plants may exhibit up- or downregulation of different genes in response to the bacteria. From the currently available literature, it appears that gene pathways involving the regulation of redox potential, ion homeostasis, leaf gas exchange, ion transport, osmolyte production, and other genes involved in stress responses such as SOS pathways, as well as transcription factors all have some control over the expression of these genes. The above pathways, or some combination of them, appear to be involved in enhanced plant growth after inoculation with ST-PGPR.

Improvements in sequencing technology and quantitative analysis of differential gene expression have improved our capabilities to analyze changes in plant gene expression in response to various signals and stresses. This includes the ability to analyze changes in the gene expression of plants inoculated with salt-tolerant bacteria compared to uninoculated plants. With time, these expression data will better elucidate the mechanisms by which ST-PGPR enhance plant growth under saline conditions. However, there are still challenges to address, which include the isolation of high-quality RNA from plant tissue and the use of a dependable computational pipeline to accurately measure differences in gene expression. The new knowledge generated as more plants and bacterial inoculants are examined will help farmers who have land affected by salinity increase their crop yield by using salt-tolerant bacterial inoculants. This will also be of great benefit for increasing agricultural productivity to feed the growing world human population, as good agricultural land is quickly decreasing.
